# Supplementary material for: Unlocking a signal of introgression from codons in Lachancea kluyveri using a mutation-selection model
Source: BMC Evol Biol. 2020 Aug 26;20:109. doi: 10.1186/s12862-020-01649-w (PMC7449078; doi:10.1186/s12862-020-01649-w)
Supplement: Supplementary file 1 — Additional file 1 Supplementary Material. Supporting Materials for Unlocking a signal of introgression from codons in Lachancea kluveri using a mutation-selection model by Landerer et al. [file 12862_2020_1649_MOESM1_ESM.pdf]

Supplementary Material

Supporting Materials for *Unlocking a signal of introgression from codons in Lachancea kluveri using a mutation-selection model* by Landerer *et al.*.

Table 1 Synonymous mutation codon preference based on our estimates of ΔM. Shown are the most likely codon in low expression genes for each amino acid in: E. gossypii, in the endogenous and exogenous genes of L. kluyveri, and in the combined L. kluyveri genome without accounting for the two cellular environments.

Amino Acid	*E. gossypii*	Endogenous	Exogenous	Combined
Ala A	GCG	GCA	GCG	GCG
Cys C	TGC	TGT	TGC	TGC
Asp D	GAC	GAT	GAC	GAC
Glu E	GAG	GAA	GAG	GAG
Phe F	TTC	TTT	TTT	TTT
Gly G	GGC	GGT	GGC	GGC
His H	CAC	CAT	CAC	CAC
Ile I	ATC	ATT	ATC	ATA
Lys K	AAG	AAA	AAG	AAA
Leu L	CTG	TTG	CTG	CTG
Asn N	AAC	AAT	AAC	AAT
Pro P	CCG	CCA	CCG	CCG
Gln Q	CAG	CAA	CAG	CAG
Arg R	CGC	AGA	AGG	CGG
Ser_4 S	TCG	TCT	TCG	TCG
Thr T	ACG	ACA	ACG	ACG
Val V	GTG	GTT	GTG	GTG
Tyr Y	TAC	TAT	TAC	TAC
Ser_2 Z	AGC	AGT	AGC	AGC

Table 2 Synonymous selection codon preference based on our estimates of $\Delta\eta$. Shown are the most likely codon in high expression genes for each amino acid in: E. gossypii, in the endogenous and exogenous genes of L. kluyveri, and in the combined L. kluyveri genome without accounting for the two cellular environments.

Amino Acid	*E. gossypii*	Endogenous	Exogenous	Combined
Ala A	GCT	GCT	GCT	GCT
Cys C	TGT	TGT	TGT	TGT
Asp D	GAT	GAC	GAT	GAT
Glu E	GAA	GAA	GAA	GAA
Phe F	TTT	TTC	TTC	TTC
Gly G	GGA	GGT	GGT	GGT
His H	CAT	CAC	CAT	CAT
Ile I	ATA	ATC	ATT	ATT
Lys K	AAA	AAG	AAA	AAG
Leu L	TTA	TTG	TTG	TTG
Asn N	AAT	AAC	AAT	AAC
Pro P	CCA	CCA	CCT	CCA
Gln Q	CAA	CAA	CAA	CAA
Arg R	AGA	AGA	AGA	AGA
Ser_4 S	TCA	TCC	TCT	TCT
Thr T	ACT	ACC	ACT	ACT
Val V	GTT	GTC	GTT	GTT
Tyr Y	TAT	TAC	TAT	TAC
Ser_2 Z	AGT	AGT	AGT	AGT

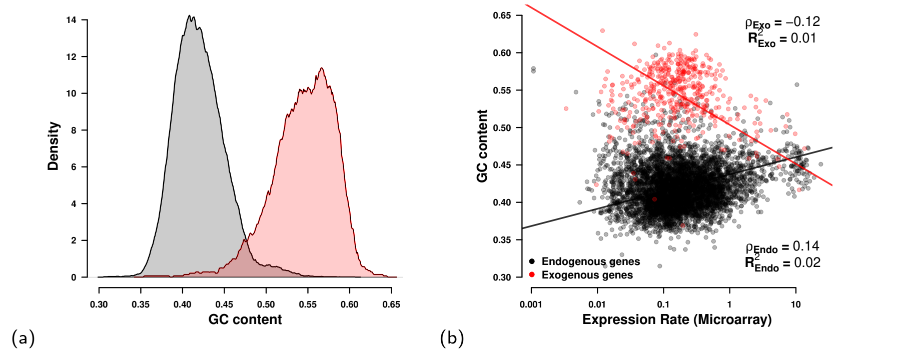

Figure 1 Endogenous and exogenous genes have distinct GC content. (a) Distribution of GC content content in the endogenous and exogenous genes. (b) Correlation of endogenous and exogenous GC content with measured gene expression. While the endogenous GC content shows a slight positive correlation with gene expression ($\rho = 0.14, p = 1.2 \times 10^{-21}$), the exogenous GC content is negatively correlated with gene expression ($\rho = -0.12, p = 0.014$).

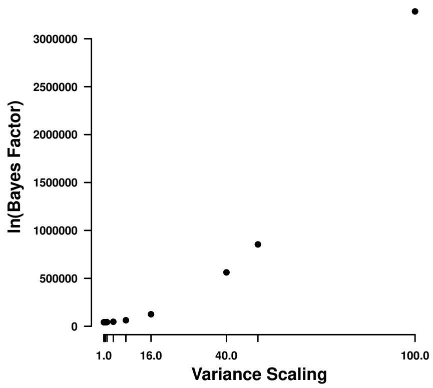

Figure 2 Influence of the variance scaling of the importance distribution on the estimated Bayes factor.

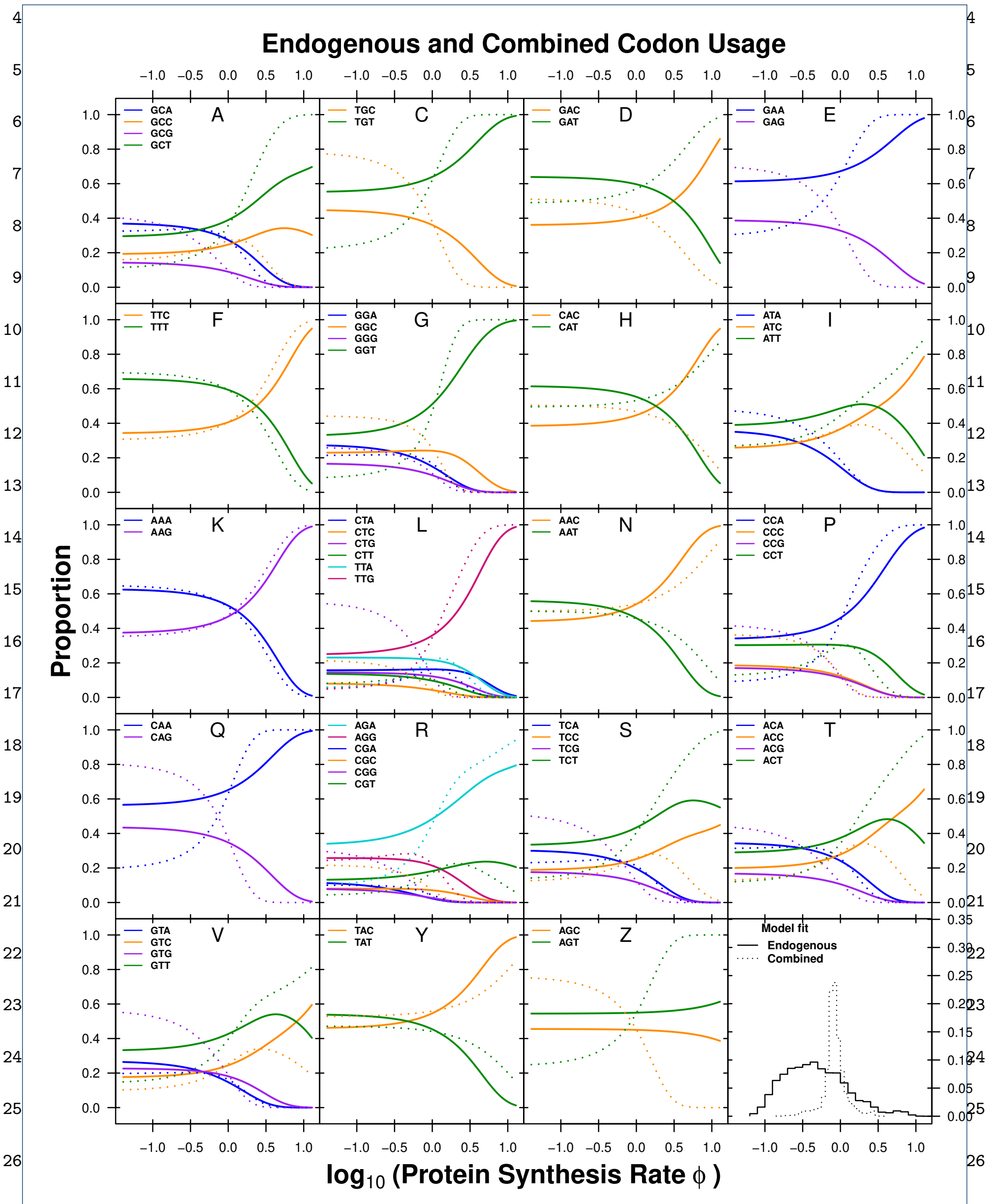

Figure 3 Codon usage patterns for 19 amino acids. Amino acids are indicated as one letter code. The amino acids Serine was split into two groups (S and Z) as Serine is coded for by two groups of codons that are separated by more than one mutation. Solid line indicates the endogenous codon usage, dotted line indicates the combined codon usage.

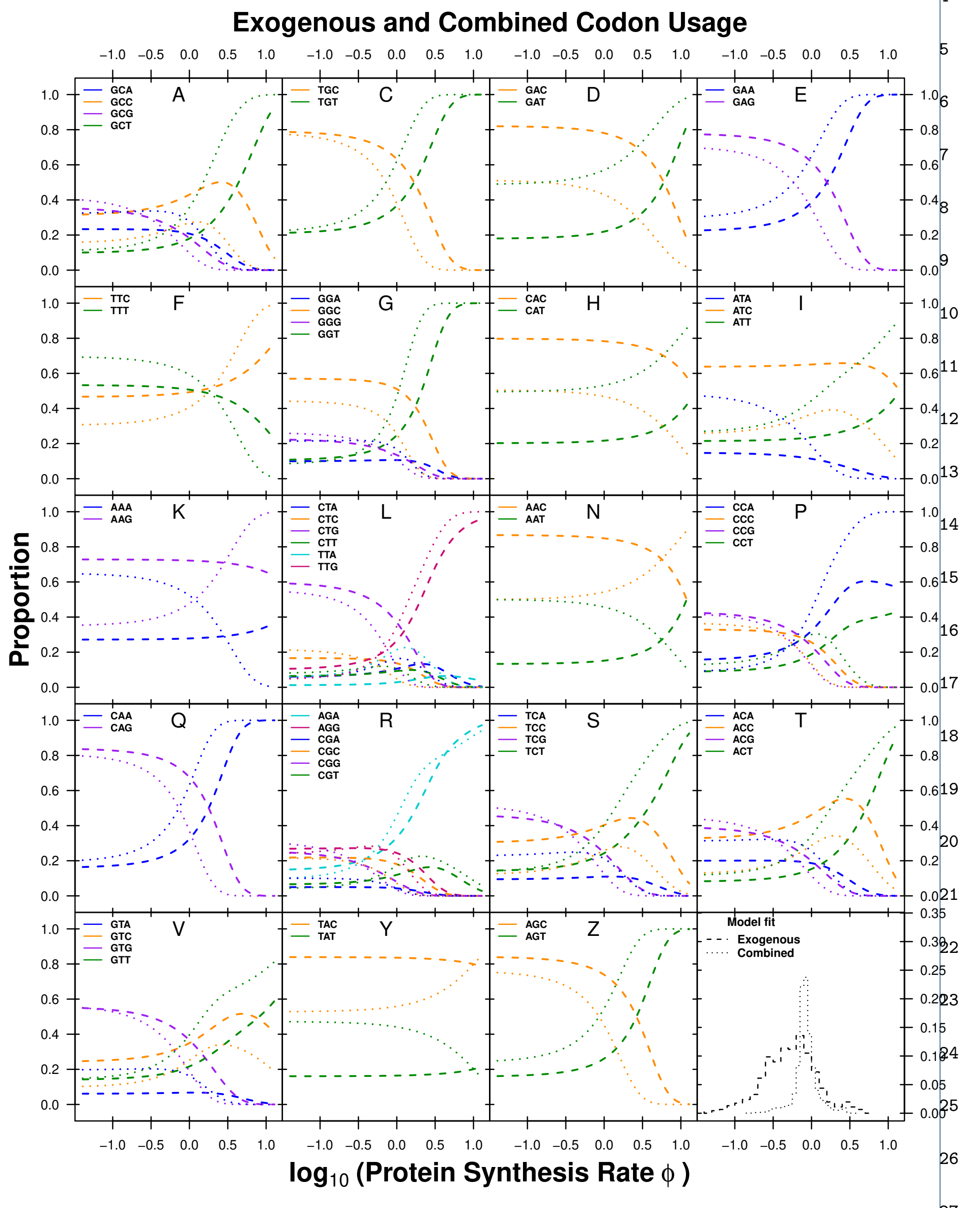

Figure 4 Codon usage patterns for 19 amino acids. Amino acids are indicated as one letter code. The amino acids Serine was split into two groups (S and Z) as Serine is coded for by two groups of codons that are separated by more than one mutation. dashed line indicates the exogenous codon usage, dotted line indicates the combined codon usage.

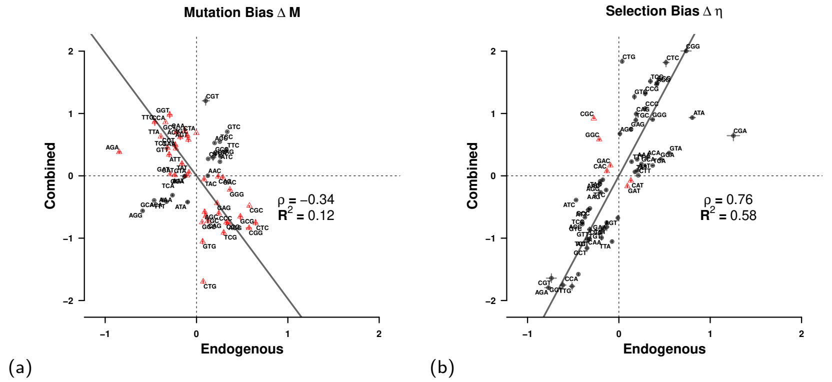

Figure 5 Comparison of (a) mutation bias ΔM and (b) selection bias $\Delta\eta$ parameters for endogenous genes and combined gene sets. Estimates are relative to the mean for each codon family. Black dots indicate ΔM or $\Delta\eta$ parameters with the same sign for the endogenous and exogenous genes, red dots indicate parameters with different signs. Black line indicates type II regression line [?]. Dashed lines mark quadrants.

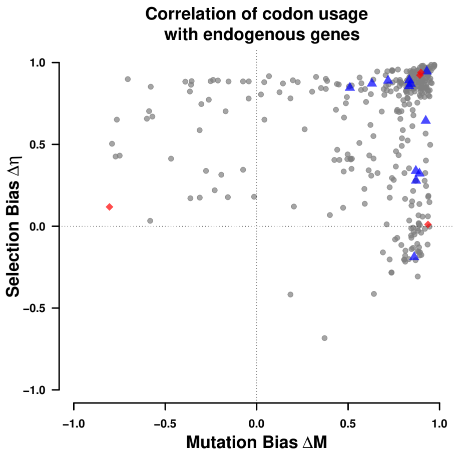

Figure 6 Correlation coefficients of ΔM and $\Delta\eta$ of the endogenous genes with 332 examined budding yeast lineages. Dots indicate the correlation of ΔM and $\Delta\eta$ of the lineages with the exogenous parameter estimates. Blue triangles indicate the Lachancea and red diamonds indicate Eremothecium lineages. All regressions were performed using a type II regression [?].

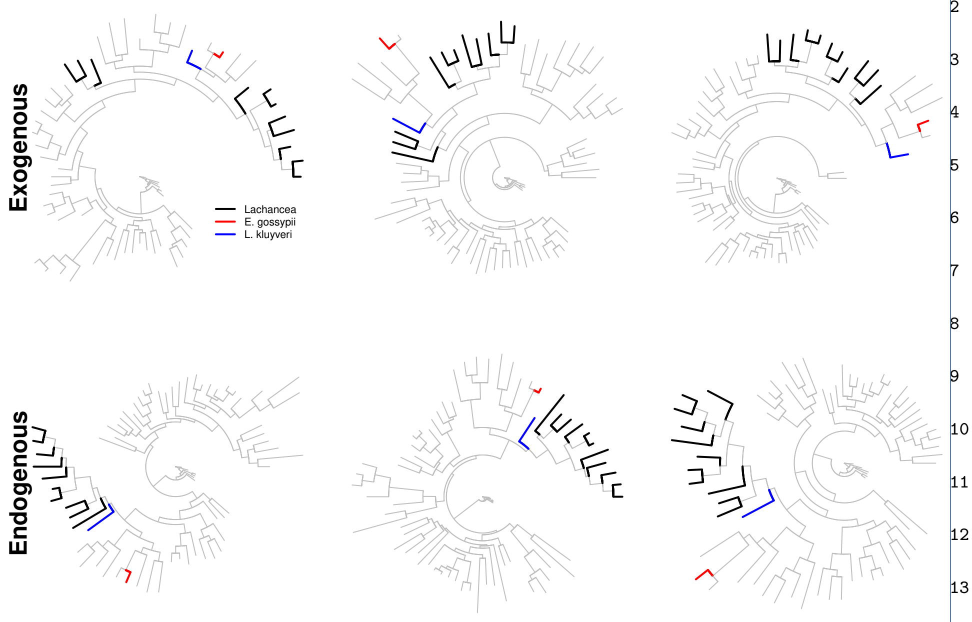

Figure 7 Gene trees illustrating the placement of *L. kluyveri* (blue) and *E. gossypii* (red) for three endogenous and three exogenous genes. The remaining Lachancea are highlighted in black. (Top row) Gene trees for three exogenous genes (from left to right: SAKL0C05742g, SAKL0C03520g, SAKL0C02376g). (Bottom row) Gene trees for three endogenous genes (from left to right: SAKL0D03960g, SAKL0G02354g, SAKL0H02552g).

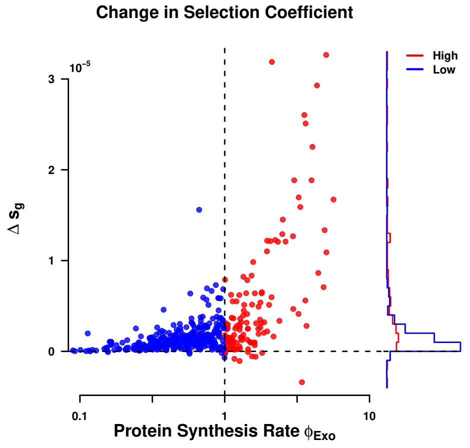

Figure 8 Total amount of adaptation estimated to have occurred between time of introgression and currently observed per gene. Vertical dashed line indicates split between high and low expression genes at $\phi = 1$. Horizontal dashed line indicates no change in selection against mismatched codon usage.

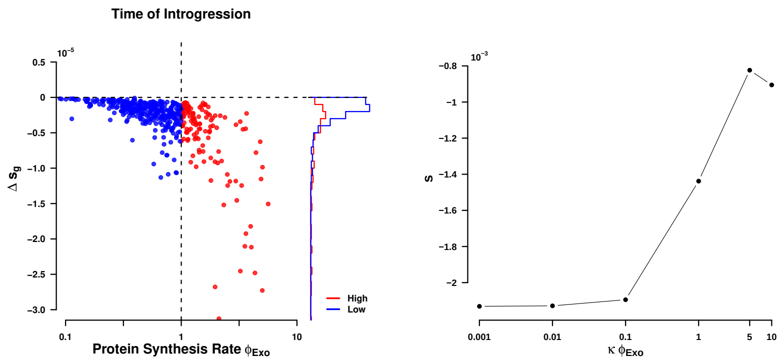

(a) (b)

Figure 9 Selection against mismatched codon usage (a) without scaling of ϕ per gene. Vertical dashed line indicates split between high and low expression genes at $\phi = 1$. Horizontal dashed line indicates neutrality. (b) Change of total selection against mismatched codon usage with scaling term κ between *E. gossypii* and *L. kluyveri*
